# Effects of Genetic Background and Environmental Conditions on Texture Properties in a Recombinant Inbred Population of an Inter-Subspecies Cross

**DOI:** 10.1186/s12284-019-0286-x

**Published:** 2019-05-09

**Authors:** Ximing Xu, Zhengjin Xu, Yuji Matsue, Quan Xu

**Affiliations:** 1 Rice Research Institute of Shenyang Agricultural University, Shenyang, 110866 China; 2 Kyushu University Global Innovation Center, Fukuoka, 812-8581 Japan

**Keywords:** Rice, Eating and cooking quality, Texture, Environmental factor, Genetic background

## Abstract

**Background:**

Eating and cooking quality have become ever more important breeding goals due to high levels of economic growth in Asia in recent decades. Cooked rice texture properties such as hardness, stickiness, and springiness are appealing to human mastication and directly reflect eating and cooking quality, and texture is strongly affected by genetic background and environmental conditions.

**Results:**

In this study, a series of recombinant inbred lines (RILs) derived from an *indica*/*japonica* cross were planted into four typical rice-cultivated areas. The relationships between the environment, texture, and genetic background of the RILs were investigated. The results showed that hardness, stickiness, and springiness strongly correlated with amylose and protein contents. Texture was strongly affected by environmental factors, which dynamically changed from the heading to the mature stage. Interestingly, the effect of environmental factors became weaker with decreasing latitude. The hardness and stickiness increased with the decrease of latitude, whereas springiness exhibited the opposite trend. The *indica* pedigree percentage did not significant correlated with hardness, stickiness and springiness. We detected 19 QTLs related to hardness, stickiness, and springiness, several of which share a similar region with a previously reported locus related to starch synthesis. Moreover, we revealed that *DEP1* might affect taste through regulating amylopectin chain length distribution.

**Conclusions:**

The present study evaluated the effects of environmental factors and genetic background to texture of cooked rice. These results provide insights into the eating and cooking quality of rice, which can be improved through sub-species crosses for different ecological conditions.

**Electronic supplementary material:**

The online version of this article (10.1186/s12284-019-0286-x) contains supplementary material, which is available to authorized users.

## Background

Grain yield and quality are major targets that are often investigated by agricultural scientists and breeders. As living standards and the economy have significantly improved during recent decades, studies have focused on rice (*Oryza sativa* L.) eating and cooking quality. Rice is one of the few major cereals that is consumed mostly in the whole grain form after cooking, tremendous efforts have been made to understand the relationship among eating and cooking quality, genetic basis and environmental condition. The eating and cooking quality is mainly affected by amylose content (AC), gel consistency (GC) and gelatinization temperature (GT) (Tian et al. 2009). Starch constitutes most of the weight of cereal grains and is the most important carbohydrate glucose polymer for humans as a dietary source of energy, the starch biosynthesis is naturally expected to affect eating and cooking quality. Starch is synthesized by four classes of enzymes, ADP-glucose pyrophosphorylase, starch synthase (SS), starch branching enzyme (BE) and starch debranching enzyme (DBE) (Ball and Morell 2003; Nakamura, 2002; Smith et al. 1997; Zeeman et al. 2010). QTL mapping and cloning provides useful information of genetic loci which related to starch biosynthesis, such (Umemoto et al. 2002; Yamanaka et al. 2004; Nakata et al. 2018). The environmental condition also strongly affected the traits of cooked rice. It has been well documented that high chalkiness of early season *indica* is mainly caused by the adverse climatic conditions during grain filling, and high temperature is the most important factor to affect chalkiness. High temperature at the filling stage accelerates filling (Umemoto et al. 1995, Yoshida and Hara 1977) and loosely-packed starch granules, results in a higher chalky occurrence in grains (Tashiro et al. 1980). Poor grain quality caused by an increase in nighttime temperatures may lead to extensive reduction in economic benefits (Lyman et al. 2013).

The eating and cooking quality are often tested by trained panelists (Takeuchi et al. 2008), and panelists testing has limitations. First, the reliability depends on the experience of the panelists. Moreover, panelists cannot evaluate plant material experimentally, such as through mapping populations and gene editing. And panelist testing is not amenable to high-throughput surveys. Cooked rice displays multiple texture properties, of which the major ones are hardness, stickiness, and springiness. Hardness and stickiness are the main factors affecting the preferences of consumers (Li and Gilbert 2018). In panelist testing, although cooked rice displays different mouthfeel among varieties, samples can be basically classified into two groups, the hardness group and stickiness group (Li et al. 2016a). Cooked rice texture is affected by a wide range of factors, such as the amylose content, postharvest processing, the method of cooking, and the *indica/japonica* subspecies (Li et al. 2016b; Champagne et al. 1998; Li and Gilbert 2018). However, the relationship among the texture of cooked rice, *indica/japonica* genetic background and environmental condition was unclear.

During recent decades, *indica*/*japonica* hybridization breeding has become an important method in China (Xu et al. 2016). The introduction of *indica* pedigrees has led to a remarkable improvement in rice production, but at the same time, a reduction in eating and cooking quality (Sun et al. 2012). In this study, we planted a series of RILs derived from crosses between *indica* variety R99 and *japonica* variety SN265 in four typical rice cultivation areas with distinctive ecological conditions. The environmental factors, the *indica* pedigree, and the texture properties were evaluated for each RIL in the four areas. Elucidating the relationships between environmental conditions, *indica*/*japonica* genetic background, and texture will provide useful information on rice breeding.

## Results

### Texture Properties of the Parent Line and RILs in the Four Areas

**Fig. 1:**
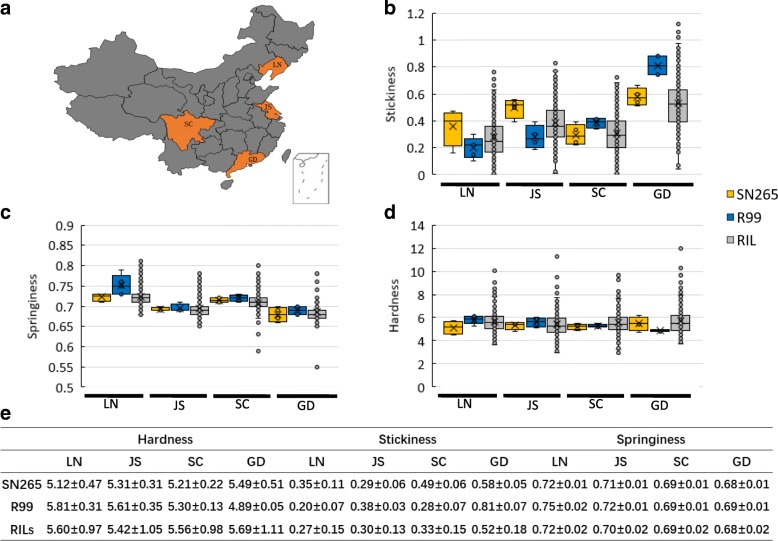
The hardness, stickiness and springiness of parent lines and RILs in four areas. **a** The location of four experimental position. **b** The stickiness of parent lines and RILs in four areas. **c** The springiness of parent lines and RILs in four areas. **d** The hardness of parent lines and RILs in four areas. **e** The data of hardness, stickiness and springiness of parent lines and RILs in four areas

We conducted a measurement of texture properties, including hardness, stickiness, and springiness, of the parent line and RILs in the four areas. The results showed that the hardness of SN265 was increased with the decrease of the cultivated area’s latitude. Meanwhile, the hardness of R99 was decreased with the decrease of the cultivated area’s latitude. In the parameter of stickiness, both SN265 and R99 showed a rising trend when the cultivated area changed from high latitude to low latitude, except the stickiness of SN265 in JS (Jiangsu province) was higher than that in SC (Sichuan province). Moreover, the stickiness of R99 was more sensitive to the change in latitude than that of SN265. Thus, SN265 had higher stickiness in LN (Liaoning province) and JS than that of R99 in SC and GD (Guangdong province), but lower than that of R99 in SC and GD. The springiness of SN265 was lower than that of R99 in all four areas. And the springiness exhibited a declining trend when the cultivated area changed from high latitude to low latitude. For the RIL population, the stickiness and hardness showed an increasing trend, whereas the springiness had a decreasing trend when the cultivated area changed from high latitude to low latitude (Fig. 1).

### Effects of Environmental Factors on Texture Properties

**Fig. 2:**
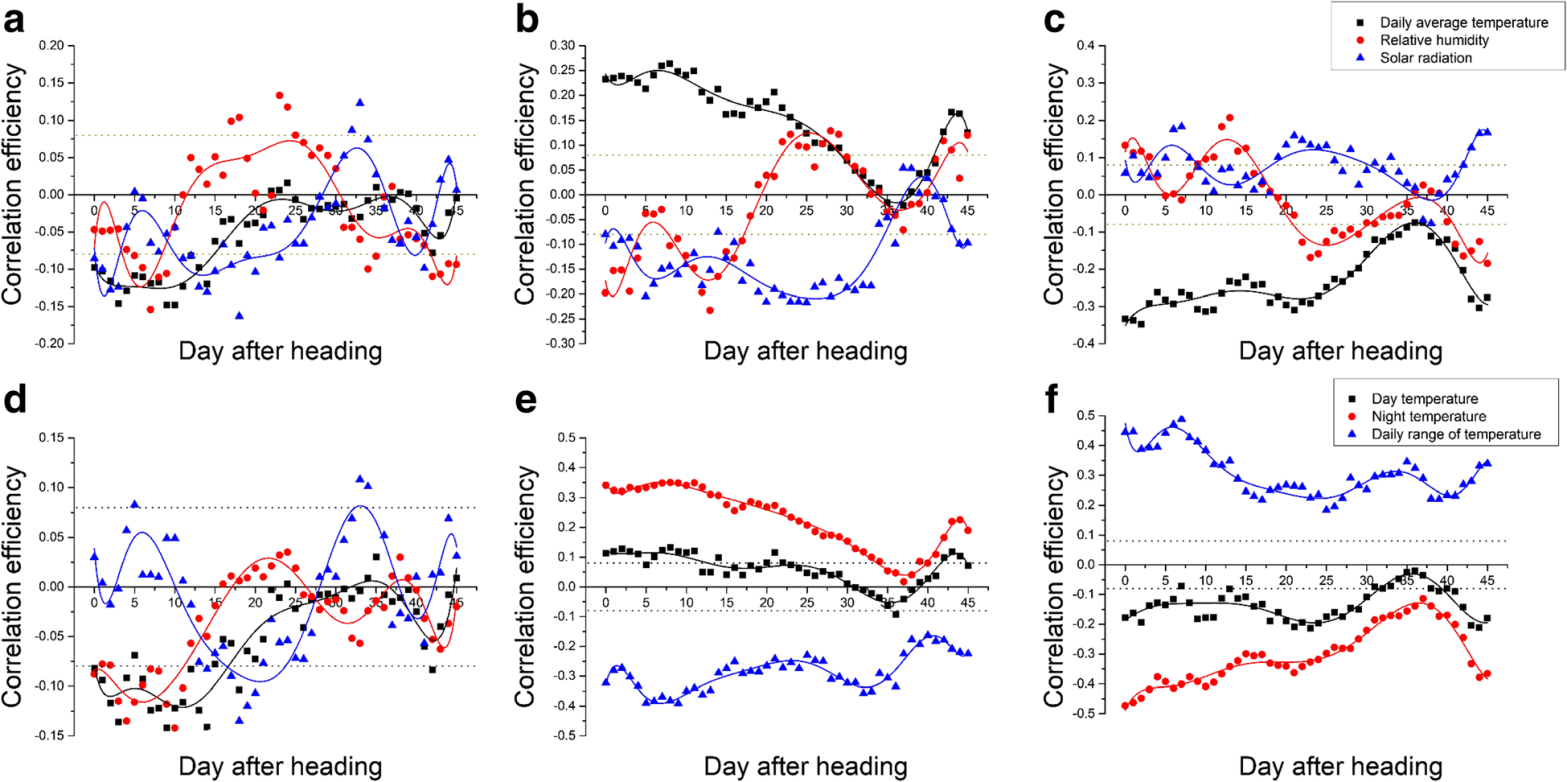
The dynamic analysis of the correlation of environmental traits to texture properties . The correlation efficiency of environmental factors (solar radiation, whole day average temperature, and relative humidity to hardness (**a**), stickiness (**b**) and springiness (**c**). The correlation efficiency of day temperature, night temperature and daily range of temperature to hardness (**d**), stickiness (**e**) and springiness (**f**). The black dot line means significant at 5% level

As the texture properties of the parent line and RILs were dramatically different among the four areas, we conducted an analysis to determine the relationship between environmental factors and texture properties. The surveyed environmental factors included solar radiation, luminous flux, light hour, air temperature, day temperature, night temperature, temperature daily range, relative humidity, day humidity, and night humidity from heading to mature (Fig. 2 and Table 1). The sowing date, heading date and mature date for each lines in four areas was shown in Additional file 1: Table S1. Overall, hardness showed a significant negative correlation to solar radiation and relative humidity. Stickiness exhibited a significant negative correlation to solar radiation, luminous flux, light hour, temperature daily range, and night humidity, but a significant positive correlation to air temperature, night temperature, and day humidity. Springiness had a significant positive correlation to solar radiation, luminous flux, light hour, temperature daily range, and night humidity, but a significant negative correlation to air temperature, day temperature, night temperature, relative humidity, and day humidity. Inside each area, we observed that the effect of environmental factors tended to become weaker with the decrease in latitude (Table 1). Then we conducted a dynamic analysis of the correlation between environmental factors and texture properties, as environmental factors are dynamic and constantly change. The results showed that the effect of these environmental factors dynamically became stronger or weaker over time (Fig. 2). In particular, relative humidity had a negative correlation with stickiness from heading to 20 days after heading, and then relative humidity showed a positive correlation with stickiness from 20 days after heading. And relative humidity had a positive correlation with springiness from heading to 20 days after heading, then relative humidity showed a negative correlation with springiness from 20 days after heading. Interestingly, the daily temperature range exhibited the opposite effect compared to the whole day average temperature, particularly in terms of stickiness and springiness.

**Table 1:**
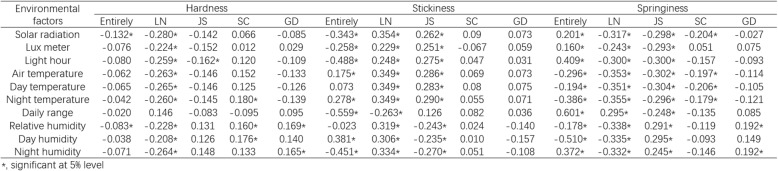
The Correlation Efficiency Between Texture properties and Environmental factors

*, significant at 5% level

### Effects of *Indica* Pedigree Percentage on Texture Properties

**Fig. 3:**
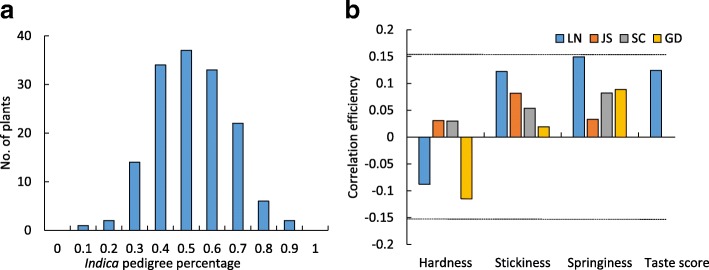
The effect of *indica* pedigree percentage to texture properties. **a** The *indica* pedigree percentage of RILs. **b** The correlation efficiency of *indica* pedigree to texture properties and taste score in LN

As texture properties vary between the two subspecies, we consequently analyzed the *indica* pedigree effects on texture properties in RILs. Our previous study had determined the *indica* pedigree of each RIL through high-throughput sequencing, and the *indica* pedigree was defined as the ratio of the number of *indica*-type SNPs to all subspecies-specific SNPs for each RIL (Li et al. 2018b). Thus, we analyzed the relationship between *indica* pedigree percentage and texture among RILs in four areas. An unexpected result in which the *indica* pedigree had a positive correlation with stickiness was observed (Fig. 3). Moreover, the correlation between *indica* pedigree and stickiness became lower with decreasing latitude. However, none of these correlation efficiencies reached significant level, which indicated the *indica* pedigree percentage barely related to texture.

### QTL Analysis of Texture Properties and Gene Function in Different Areas

**Fig. 4:**
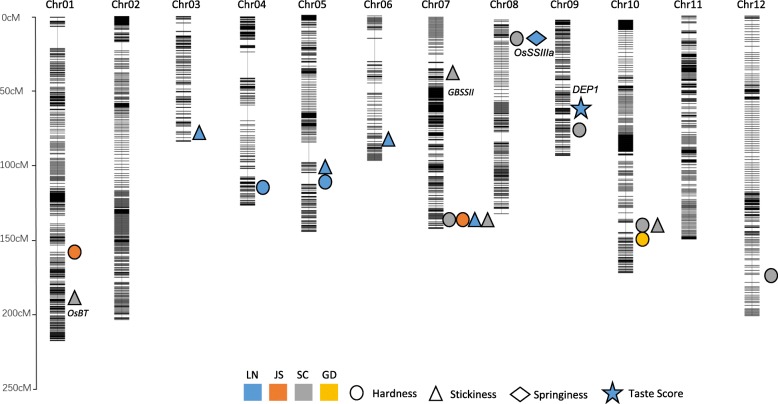
The results of QTL analysis in four areas

**Fig. 5:**
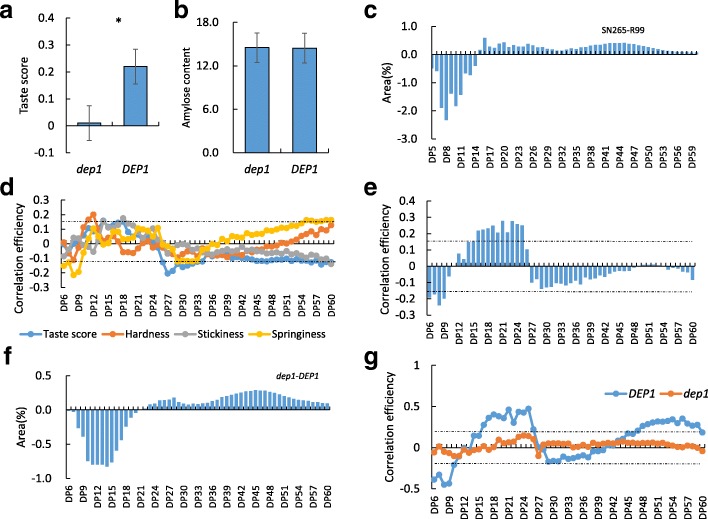
Comparison and difference in amylopectin chain length distribution. **a** The comparison of taste score between *dep1* carrying lines and *DEP1* carrying lines. **b** The comparison of amylose content between *dep1* carrying lines and *DEP1* carrying lines. **c** The comparison in amylopectin chain length distribution between SN 265 and R99. **d** The correlation efficiency of amylopectin chain length to taste score, hardness, stickiness and springiness. **e** The correlation efficiency between amylopectin chain length and *indica* pedigree percentage. **f** The comparison of amylopectin chain length distribution between *dep1* carrying lines and *DEP1* carrying lines. **g** The correlation efficiency between *indica* pedigree percentage and amylopectin of *DEP1* carrying lines and *dep1* carrying lines. * and black dot line indicate significant at 5% level

A QTL analysis was conducted using the texture property data and the bin-map that we published previously (Li et al. 2018b). A total of 19 QTLs located on chromosomes 1, 3, 4, 5, 6, 7, 8, 9, 10, and 12 were detected in the present study. Nine QTLs corresponding to hardness were detected on chromosomes 1, 4, 5, 7, 8, 9, 10, and 12; seven QTLs related to stickiness were detected on chromosomes 1,3 5, 6, 7, and 10; and only one QTL was detected on chromosome 8. Only the QTLs on chromosomes 7 and 10 for hardness and the QTLs on chromosome 7 for stickiness were detected in more than one area (Fig. 4 and Table 2). QTLs on chromosomes 5, 7, and 10 were detected for both hardness and stickiness, and a QTL on chromosome 8 was detected for both hardness and springiness. These results indicated that these QTL clusters may be controlled either by one gene with pleiotropy or by a group of closely linked genes. As *Waxy/GBSSI* which impacts the overall cooking and eating quality was not detected in the present QTL analysis. We conducted the genetic background diagnosis of the parent lines. The results showed that both SN265 and R99 carried *WxB* type allele at *Waxy/GBSSI* locus. The detail of the genetic background diagnosis was shown in Additional file 2: Table S2. As the quality trait are easily affected by environmental factors, we also conducted the QTL analysis using the data collected in 2015 (Additional file 3: Table S3). The results showed the QTLs on Chr.1, Chr.7 and Chr.8 were detected in both 2015 and 2016. We further conducted the QTL analysis of taste score in LN, interestingly, only one locus (*qTS9*) was detected (Fig. 4). The candidate gene was mapped to a 43-kb interval in block 19,448. Five annotated genes were present in this block, and the sequence analysis showed that a replacement of a 637-bp fragment in the middle of the 5th exon at *DENSE AND ERECT PANICLE 1* (*DEP1*) in SN265 (Huang et al. 2009). The detail of sequence analysis was listed in Additional file 4: Figure S1. Then we surveyed expression pattern of annotated gene, only *DEP1* showed expression preference at panicle (Additional file 4: Figure S1). *DEP1* has been previously reported to be a pleiotropic major QTL responsible for grain number and panicle architecture. We conducted a co-segregation analysis of *DEP1*, which showed that plants harboring the SN265-type allele of *dep1* exhibited lower taste score than that of lines carrying R99 type *DEP1* (Fig. 5a). As our previous study had generated the *dep1* mutant using CRISPR/Cas9 gene editing technology (Li et al. 2018b), we selected the mutants without Cas9 protein, and compared the taste score between mutants and WT. The mutants exhibited significant lower taste score compare to that of WT (Additional file 4: Figure S1). Thus, we concluded the *DEP1* locus responses for the diversity of eating quality among parents and RILs. As the *qTS9* was not detected in the QTL analysis of hardness, stickiness and springiness, we hypothesized that *qTSq* affect taste score through other pathway. No significant difference of amylose content was detected between the *DEP1* harboring lines and *dep1* carrying lines (Fig. 5b). Since varieties with similar amylose content also showed difference in eating and cooking quality due to the diversity of amylopectin chain length distribution. Thus, we tested the amylopectin chain length distribution of parent lines and RILs. The results showed that R99 was markedly enriched in chain length with DP5-DP14 compare to the R99, and SN265 was enriched in DP15-DP60 compare to the R99 (Fig. 5c). The amylopectin chain length distribution had diverse correlation to taste score, hardness, stickiness and springiness (Fig. 5d). The *indica* pedigree percentage had a significant negative correlation to DP6-DP10, whereas a significant positive correlation to DP14-DP24 (Fig. 5e). Then we conducted the QTL analysis of amylopectin chain length distribution. As the Additional file 5: Table S4 shown, the block 199,448 of *qTS9/DEP1*was detected corresponding for DP10–12, DP13–15, DP16–18 and DP37–60. We further compare the amylopectin chain length distribution between *DEP1* carrying lines and *dep1* carrying lines. The results showed that the *DEP1* carrying lines was markedly enriched in DP6-DP18 (Fig. 5f). Moreover, the correlation efficiency between amylopectin chain length distribution and *indica* pedigree percentage in *DEP1* carrying lines was extremely significant than that of *dep1* carrying lines (Fig. 5g).

**Table 2:**
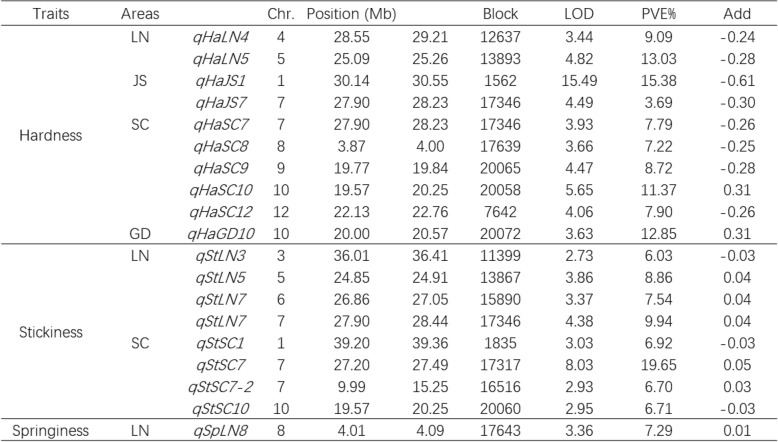
QTL mapping for Texture properties of the RIL population

### Relationship Between Texture Properties, Yield Components, and Quality Traits

**Fig. 6:**
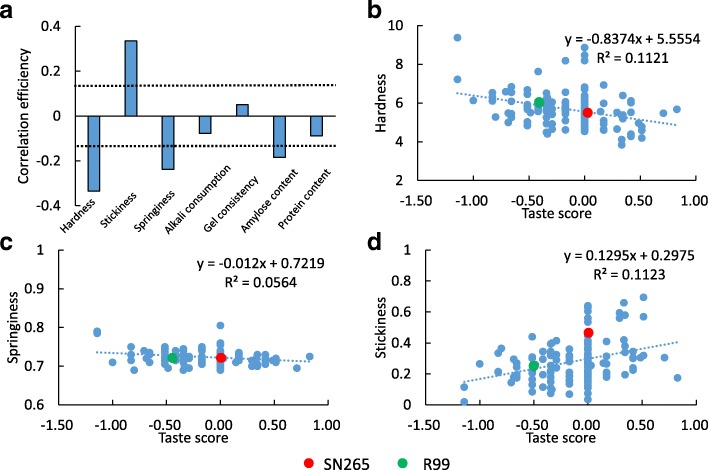
The relationship of taste score to hardness, stickiness and springiness in LN. **a** The correlation efficiency of taste score to other quality traits. **b** The correlation efficiency of taste score to hardness. **c** The correlation efficiency of taste score to springiness. **d** The correlation efficiency of taste score to stickiness. The black dot line means significant at 5% level

We investigated the yield components, quality traits of RILs in four areas, the data was shown in Additional file 6: Table S5. Then, we conducted a relationship analysis of texture properties with yield components and quality traits. When we combined the data from the four areas, hardness showed a significant positive correlation to 1000 grain weight, but a significant negative correlation to grain number per panicle and spikelet density. Stickiness exhibited a significant negative correlation to grain number per panicle, setting rate, panicle number, and finally yield potential. Springiness showed a significant positive correlation to setting rate, panicle number, and yield potential. However, diverse relationships were observed inside each area. In LN, hardness was significantly positively correlated to 1000 grain weight. In SC, hardness had a significant negative correlation to grain number per panicle and spikelet density. Springiness showed a significant negative correlation to spikelet density. No significant correlation was observed in JS and GD (Table 3). Then we analyzed the correlation between texture properties and other quality-related traits, including brown rice ratio, milled rice ratio, head rice ratio, chalkiness rice ratio, chalkiness level, grain shape (the ratio of grain length to grain width), alkali consumption, gel consistency, amylose content, and protein content (Table 4). Overall, hardness showed a significant negative correlation to head rice ratio, but a significant positive correlation to amylose and protein contents. Stickiness exhibited a significant negative correlation to brown rice ratio, milled rice ratio, and amylose content, but a significant positive correlation to protein content. Springiness had a significant positive correlation to brown rice ratio, milled rice ratio, head rice ratio, and amylose content, but a significant negative correlation to alkali consumption and protein content. Diverse correlations between texture properties and other qualities were observed inside each area. The general trends were that increased hardness reduced the head rice ratio, and increased springiness improved the brown rice ratio and milled rice ratio. Increased protein content increased both hardness and springiness. Increased amylose content increased hardness but decreased stickiness. Then we analyzed which quality parameters had the closest relationship with human testing. The results showed that hardness, stickiness, and springiness were the top three parameters that had the closest relationship with human testing (Fig. 6).

**Table 3:**
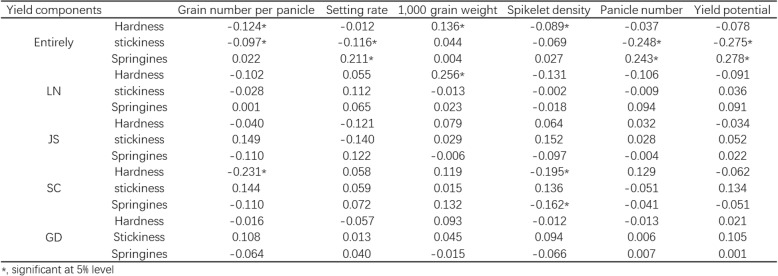
The correlation coefficient of texture properties and yield components

*, significant at 5% level

**Table 4:**
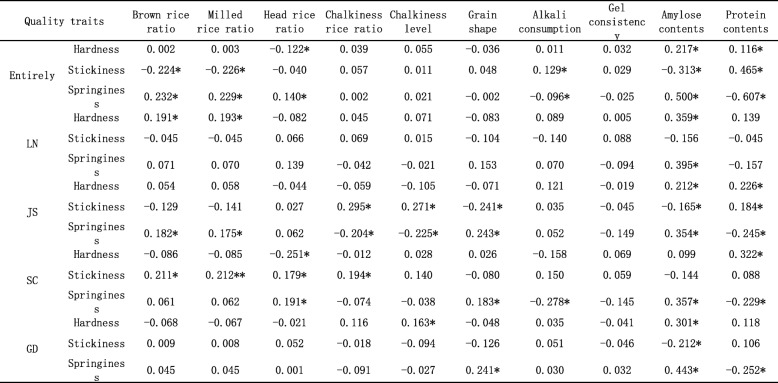
The correlation coefficient of texture properties and quality properties

*, significant at 5% level

## Discussion

Recent molecular studies have identified a tremendous number of genes/QTLs related to grain yield in rice (Huang et al. 2009; Jiao et al. 2010; Xing and Zhang 2010). However, research into grain quality, especially eating and cooking quality, has lagged due to a lack of efficient methods to evaluate these qualities. Previous studies demonstrated that the eating and cooking quality is mainly affected by amylose content, gel consistency, and gelatinization temperature (Tian et al. 2009; Nakata et al. 2018). Besides amylose content, gel consistency, and gelatinization temperature, rapid visco analyzer (RVA) and differential scanning calorimetry (DSC) have commonly been used in determining the physicochemical parameters (Cheng et al. 2005). Our study found that hardness, stickiness, and springiness, which can be easily evaluated, had a closer relationship with eating and cooking quality than the traits mentioned above. Agronomic traits, including eating and cooking quality, are determined by interactions between the genotype and the environment. This study showed that environmental factors may have a stronger effect on texture properties than genetics, as the parent line SN265 had a higher stickiness value than that of R99 in LN and JS, but lower than that in SC and GD. Thus, the genes/QTLs that regulate texture properties may only adapt to a particular cultivation area, which may make research on eating and cooking quality even more difficult.

Differences in eating and cooking quality between *indica* and *japonica* have historically been recognized. Various differences in texture properties have been detected between subspecies, and cooked *japonica* rice is considered to be more sticky than cooked *indica* rice. In general, *japonica* has the advantage in eating and cooking quality compared to *indica*, which led us to hypothesize that the higher the *indica* pedigree percentage, the lower the eating and cooking quality. However, our study showed that the *indica* pedigree percentage was barely correlated to stickiness, springiness and hardness. Taken together, the differences in eating and cooking quality between *indica* and *japonica* may not come mainly from the genetic background. The quality traits including rice textures are easily affected by environmental factors, our QTL analysis only found several locus which could be detected at more than one area or both in 2015 and 2016 (Table 2 and Additional file 3: Table S3). Given that starch comprises approximately 90% of a rice grain, genes involved in starch biosynthesis are naturally expected to affect eating and cooking quality. *OsSSIIIa/Flo5* is involved in starch synthesis in rice, and mutants of the gene are characterized by an endosperm with a loosely packed central portion exhibiting a floury-like phenotype (Ryoo et al. 2007). *OsDPE1* has been reported as a disproportionating enzyme in rice that can disproportionate maltotriose to produce glucose and maltopentaose, and thus shares the defining behavior of a D-enzyme (Hwang et al. 2016). The present study detected a pleiotropic QTL corresponding to hardness and stickiness in a similar region as *OsDPE1* at LN, JS and GD in both 2015 and 2016, which indicated that *OsDPE1* may affect eating and cooking quality by regulating texture properties. We noticed that the *Waxy/ GBSSI* which impacts the overall cooking and eating quality was absence in the QTL analysis (Yamanaka et al. 2004; Tian et al. 2009). The genetic background diagnosis showed that both parents harboring the *Wxb* type allele at *Waxy/GBSSI* locus. As it is well known that *Waxy/GBSSI* was the major gene affects grain quality, these parent lines share same *Waxy/GBSSI* allele might help us know more genes affecting grain quality besides *Waxy/GBSSI.* Interestingly, we found a QTL (*qTS9*) corresponding to taste score was synonymous to *DEP1*. The further analysis demonstrated that *DEP1* might affect taste score through regulating amylopectin chain length distribution. As erect panicle type varieties were considered to be only mediocre eating quality (Xu et al. 2016), the participation of *DEP1* in amylopectin chain length distribution might explain the mediocre eating quality of erect panicle type varieties, and the lines carrying *dep1* allele showed insensitivity to the change of *indica* pedigree percentage in amylopectin chain length distribution. Then we scan the lines harboring *dep1* allele, found line 26, 41 and 48 was enriched in DP6–14 compare to SN265. These materials might provide useful germplasm to improve the eating and cooking quality of erect panicle varieties.

As plants grow in a constantly changing environment, plants with different growth periods are subjected to variations in environmental conditions even when they are cultivated in the same area. Thus, investigations on the impact of environmental factors on crop production of specific plants are warranted. This study determined that the effects of environmental factors on texture properties dramatically changed from the heading to the mature stage. These results suggest that appropriate adjustments to the sowing date should be made to cater to or avoid the corresponding advantages or disadvantages of specific environmental conditions.

## Conclusions

Eating and cooking quality is an important trait in rice breeding and mainly evaluated through sensory testing. The present study the texture properties such as hardness, stickiness and springiness of cooked rice might directly reflect the eating and cooking quality of rice. The texture of cooked rice was strongly affected by ecology condition, and barely affected by the *indica* pedigree percentage. And the *DEP1* might affect eating quality through regulating the amylopectin chain length distribution. Combined with our previous studies, we suggested that the introgression of *indica* pedigree could increase the yield without quality penalty imposed.

## Materials and Methods

### Plant Material

A total of 155 RILs derived from a cross between ‘Shennong265’ (*Oryza sativa* L. ssp. *japonica*) and ‘R99’ (*Oryza sativa* L. ssp. *indica*) were used in this study. This RIL population was developed from a single-seed descendant that had been inbred for over 10 generations. Field experiments were conducted in four typical rice cultivating areas: the Rice Research Institute of Shenyang Agricultural University (SY) (N41°, E123°), the sub-base of the China National Hybrid Rice R&D Center in Jiangsu Province (JS N32°, E120°), the Academy of Agricultural Sciences of Sichuan Province (SCN32°, E104°), and the Agricultural Genomics Institute at Shenzhen (SZN22°, E114°) for two growing seasons during 2015–2016. As the both data of 2 years showed similar trends, the data of 2016 was used in the analysis. The cultivation method and field management were described in our previous report (Li et al. 2018a).

### Measurement of Texture Properties, Yield Components, and Other Quality Traits

The measurement of texture properties was conducted as follows: the hulled grains were milled at a 90% milling rate and stored at 5 °C until analysis (Matsue et al. 1991). Texture properties were examined at the China-Japan Joint Research Center on Palatability and Quality of Rice in Tianjin Agricultural University. The white rice was washed several times and soaked for 40 min before cooking in an electric rice cooker (Cooking steamer; SATAKE Co. Ltd., Japan) at a ratio of white rice to water of 1:1.35(*w*/*v*). Texture properties (hardness, stickiness and springiness) of cooked rice were measured with a hardness-stickiness texture analyzer (RHS1A; SATAKE Co. Ltd., Japan). All samples were analyzed with two biological replicates. At least three technical replicates were analyzed for each biological replicate and expressed as an average. The survey of yield components and other quality traits was conducted as described in our previous study (Xu et al. 2014; Fan et al. 2017). Starch was extracted from the flour and debranched following a method described by Hasjim et al. (2010) with modifications. The debranched starch was labeled using 8-aminopyrene-1,3,6,trisulfonic acid following a procedure described by Wu et al. (2014) and then separated with a carbohydrate separation buffer (Beckman-Coulter, Brea, CA, USA). The chain length distribution (CLD) of debranched amylopectin was characterized using a PA-800 Plus FACE System (Beckman-Coulter), coupled with a solid-state laserinduced fluorescence detector and an argon-ion laser as the excitation source. Samples were analyzed in duplicate.

### Survey of Environmental Factors

The environmental factors were measured using iMETOS (Pessl Instruments GmbH, Weiz, Austria) at the four areas. We surveyed three main environmental factors: light, which was subsequently divided into solar radiation, luminous flux, and light hours; temperature, which included the whole-day average air temperature, day air temperature, night air temperature, and daily temperature range; and humidity, which included the whole-day average relative humidity, day relative humidity, and night humidity. Details of the measurement methods were described in our previous study (Li et al. 2018a).

### Data Analysis

We sequenced the RILs and parent lines on an Illumina HiSeq2500 platform to determine the *indica* pedigree percentage and conduct QTL analysis. The details were described in our previous study (Li et al. 2018b). Statistical analysis was performed using Duncan’s new multiple range method and Bivariate Correlations of Pearson correlation coefficients with SPSS 23.0 (IBM, USA). In addition, QTL analysis was performed using QTL IciMapping V4.1(Institute of Crop Science of CAAS, PR China). Tables were built using Excel 2018 (Microsoft. Co., USA), and Figures were created using Origin 9.0 Professional (Origin Lab. Co., USA).

## Additional Files

Additional file 1: **Table S1.** The sowing, heading and mature date of RILs in four areas. (XLSX 27 kb)

Additional file 2: **Table S2.** The quality related genetic background diagnosis of parent lines. (XLSX 14 kb)

Additional file 3: **Table S3.** QTL mapping for Texture properties of the RIL population in 2015. (XLSX 10 kb)

Additional file 4: **Figure S1.** The fine mapping of dep1. (a) The fine mapping of DEP1 using the data of amylopectin chain length distribution. (b) The sequence comparison of annotated genes in block 19,948. (c) The express preference of annotated genes in block 19,948. (d) The image of *DEP1* mutant generated by CRISPR/Cas9 gene editing technology, and the taste score of mutant and WT (Sasanishiki). *, significant at 5% level. (XLSX 1871 kb)

Additional file 5: **Table S5.** The QTL analysis of amylopectin chain length distribution. (XLSX 9 kb)

Additional file 6: **Table S6.** The yield and quality traits of RILs in four areas. (XLSX 143 kb)
